# Dendronized Gelatin-Mediated Synthesis of Gold Nanoparticles

**DOI:** 10.3390/molecules27186096

**Published:** 2022-09-18

**Authors:** Yan Ding, Xiacong Zhang, Wen Li, Afang Zhang

**Affiliations:** 1 International Joint Laboratory of Biomimetic and Smart Polymers, School of Materials Science & Engineering, Shanghai University, Shanghai 200444, China; 2 Institute of Chemistry and Materials Science, Huaibei Normal University, Dongshan Road 100, Huaibei 235000, China

**Keywords:** gelatin, gold nanoparticles, dendronized polymer, thermoresponsiveness, microconfinement

## Abstract

Thermoresponsive dendronized gelatins (**GelG1**) or gelatin methacrylates (**GelG1MA**) were used as precursors to modulate the efficient reduction of Au(III) to form stable gold nanoparticles (AuNPs) through UV irradiation. These dendronized gelatins were obtained through the amidation of gelatin or gelatin methacrylates with dendritic oligoethylene glycols (OEGs). Crowded OEG dendrons along the gelatin backbones create a hydrophobic microenvironment, which promotes the reduction of Au(III). Gelatin backbones act as ligands through the electron-rich groups to facilitate the reduction, while the dendritic OEGs provide shielding effects through crowding to form a hydrophobic microenvironment, which not only enhances the reduction but also stabilize the formed AuNPs through encapsulation. The effects of dendron coverage on the dendronized biomacromolecules and their thermoresponsiveness on the reduction kinetics were examined. Dendronized gelatin/AuNPs hydrogels were further prepared through the in situ photo-crosslinking of **GelG1MA**. The modification of natural macromolecules through dendronization presented in this report facilitates a novel platform for the environmentally friendly synthesis of noble metal nanoparticles, which may form a new strategy for developing smart nano-biosensors and nano-devices.

## 1. Introduction

The modification of natural macromolecules has attracted more and more attention in recent years to create novel environmentally friendly materials, which not only inherit excellent biofunctions from biomacromolecules, but also can be endowed with new properties through functional modification [1,2,3,4]. Natural macromolecules carry abundant functional groups, such as hydroxyl, amine and carboxyl groups, which can be readily used for chemical modification. In recent years, various strategies have been applied for the modification of biomacromolecules, aimed at different functions and properties. Modified natural macromolecules show advantages in stability, safety, responsibility, and sustainability, and have been widely used in fields from smart materials to biomedicine [5].

Gold nanoparticles (AuNPs) exhibit unique physical and chemical properties, and have attracted extensive attention continuously. They have been applied in various fields, such as optics, electronics, catalysis [6] and liquid crystal composites [7], as well as in drug delivery [8], biological imaging [9], and cancer treatment [10]. AuNPs can be prepared through the reduction of Au(III) by various agents, such as alcohols, amines, carboxylic acids or borohydride. Since the nanoparticles formed tend to aggregate in aqueous solutions, the effective strategy to avoid this is to protect them by stabilizers, such as thiols, polymers or polyelectrolytes [11]. For this purpose, many polymer stabilizers, such as microgels, dendritic macromolecules, hydrogels and latex particles, have been applied. Polymer stabilizers can control the reduction rate and sizes of formed nanoparticles [12]. Traditional synthesis methods usually use toxic chemicals, which may cause some pollution to the environment. In addition, the residues of chemical substances may affect the biocompatibility of the prepared particles and limit their biomedical applications. It is safer and greener to use environmentally friendly reductants and stabilizers and mild experimental conditions [13]. Therefore, significant efforts have been given to the development of clean and environmentally friendly methods for synthesis of AuNPs. For this purpose, biomacromolecules, including polysaccharides, protein and peptides, have been successfully applied to synthesize and stabilize AuNPs [14]. These biomacromolecules can protect AuNPs after their formation through steric hindrance, and prevent them from further aggregation [15,16,17]. For example, red highly fluorescent gold nanoclusters were prepared by bovine serum albumin (BSA) at physiological temperature (37 °C) [18]. Pepsin-mediated gold nanoclusters were reported, showing blue, green and red fluorescent emission through pH modulation [19]. AuNPs were also synthesized by chitosan (CS)/poly (methacrylic acid) hybrid semi-interpenetrating nanogel [20]. Lysosome–dextran protein polysaccharide nanogel was used to stabilize AuNPs and the composite material was applied for drug delivery and biological imaging [21].

As one intriguing class of biomacromolecules, gelatin exhibits excellent biocompatibility, biodegradability and low antigenicity. Furthermore, it is abundant and quite cheap. It has been recognized as a generally recognized safe (GRAS) material by the U.S. Food and Drug Administration (FDA) [22]. Gelatin is usually used as a stabilizer in the synthesis of AuNPs. For example, AuNPs can be synthesized in the matrix of gelatin through reducing tetrachloroauric acid with sodium citrate [23]. Hybrid nanogels containing AuNPs were prepared in the presence of protein polyion complexes (PICs) through cross-linking from gelatin and two proteins: horseradish peroxidase (HRP) and lactoferrin (LTF) [13]. These nanogels exhibited colloidal stability and metal-enhanced luminescence/fluorescence (MEL/MEF), which are promising for optically enhanced diagnosis and other therapeutic applications. However, these PIC NPs were not stable enough and were found to disassemble after dilution [24].

**Figure 1:**
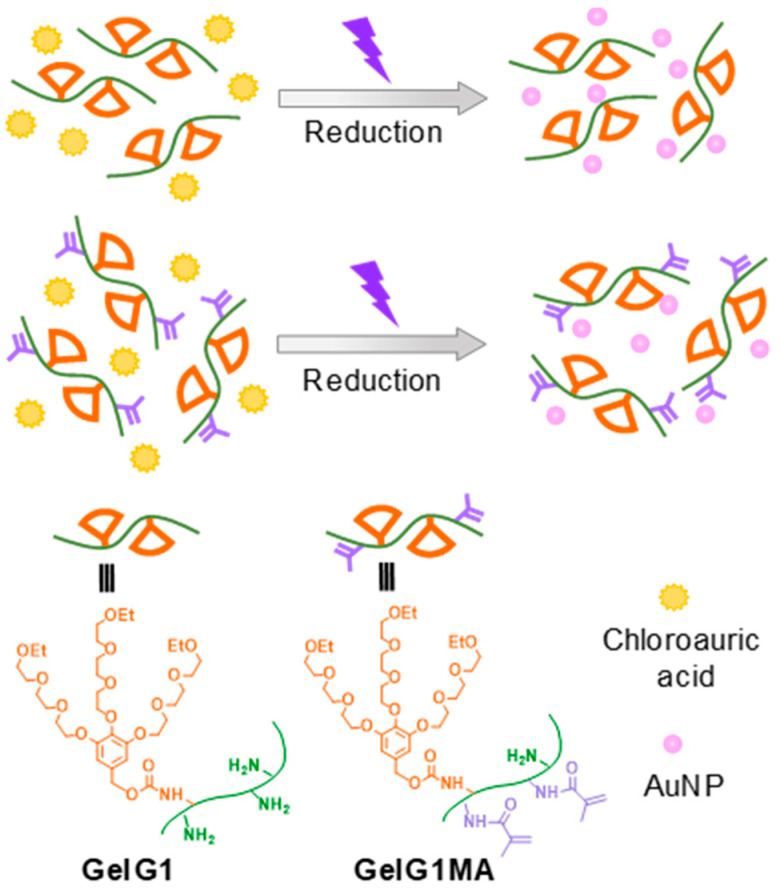
Illustration of synthesis of AuNPs in the matrix of dendronized gelatins (**GelG1**s) and dendronized gelatin methacrylates (**GelG1MA**s) triggered by UV irradiation.

Currently, most synthesized AuNPs have a single function, and regulating their functionalization is not always feasible. Furthermore, the stability of AuNPs is not always satisfactory to meet the application requirements. Therefore, developing a simple and environmentally friendly method for the synthesis of multifunctional AuNP composites with high stability is important. Recently, we found that the dendronization of a polymer with dendritic oligoethylene glycols (OEGs) can afford the polymer characteristic of thermoresponsiveness with a heterogeneous dehydration process [25] and simultaneously create a confined microenvironment to modulate the physical properties of guest moieties [26] or even guest biomacromolecules [27]. Through a similar strategy, the dendronization of chitosan can afford the modified biomacromolecules thermoresponsiveness, showing microconfinement to promote the formation of silver nanoparticles (AgNPs) [28]. By using the same method, we also prepared a class of thermoresponsive dendronized gelatins (**GelG1**s) [29]. Here, we report on the synthesis of AuNPs by using these dendronized gelatins as the matrix, without any additional reducing agent (Figure 1). **GelG1**s carry dendritic OEGs as pendants, which not only provide characteristic thermoresponsiveness, but also endow these modified gelatins’ microconfinement [29]. Furthermore, **GelG1MA**s carrying additional methacrylate moieties were prepared, allowing the preparation of corresponding thermoresponsive hydrogels through in situ photo-crosslinking. AuNPs were prepared through in situ reduction in the matrix of these dendronized gelatins, assisted by ultraviolet irradiation. The effects of topological structures and the thermoresponsiveness of these dendronized gelatins on the reduction of AuCl_4_^−^ into AuNPs were investigated. The encapsulation and stability of AuNPs within the dendronized gelatins were examined.

## 2. Results and Discussion

### 2.1. Synthesis and Characterization of GelG1 and GelG1MA

**Figure 2:**
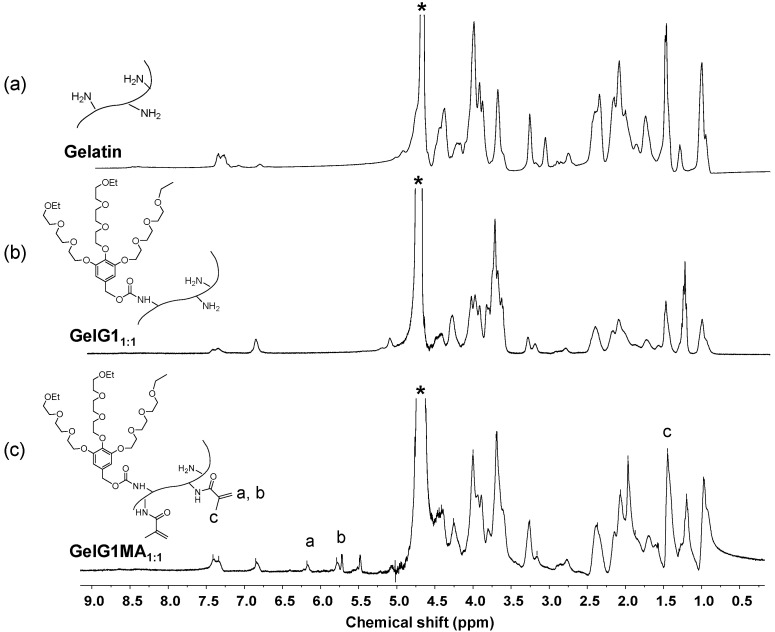
^1^H NMR spectra of naked gelatin (**a**), **GelG1_1:1_** (**b**) and **GelG1MA_1:1_** (**c**) in D_2_O. Solvent signals are marked with asterisk (*).

Dendronized gelatins were prepared through amidation with dendritic OEGs, as described previously [29]. In order to examine the dendritic effects on the modulation of the reduction of AuCl_4_^−^ into AuNPs, two dendronized gelatins—**GelG1_15:1_** and **GelG1_5:1_**—with an OEG dendron substitution degree of 86.7% and 54.4%, respectively, were synthesized and compared to the naked gelatin. In addition, **GelG1MA_1:1_** with methacryloyl pendants was synthesized from **GelG1_1:1_** (Scheme S1), allowing for the formation of dendronized gelatin hydrogels via photo-crosslinking. The substitution degrees of OEG dendron and methacryloyl moiety in **GelG1MA_1:1_** were 45.6% and 48.5%, respectively. The structures of the dendronized gelatins were confirmed with ^1^H NMR spectroscopy. Figure 2 shows the assembled ^1^H NMR spectra of naked gelatin, **GelG1_1:1_** and **GelG1MA_1:1_**. Compared to the unmodified gelatin and **GelG1_1:1_**, two new signals (a and b) at δ 6.19 ppm and 5.8 ppm were observed, corresponding to the protons from the methacryloyl moiety. The intensity of signal c at δ 1.45 ppm, which corresponds to the methyl group from the methacryloyl moiety, increased obviously after modification. This confirms that methacryloyl groups were successfully grafted to the gelatin. These dendronized gelatins exhibit characteristic thermoresponsive properties, and the cloud points (T_cp_s) for **GelG1_15:1_**, **GelG1_5:1_** and **GelG1MA_1:1_** at a concentration of 0.5 wt% were found to be 35.7 °C, 45.8 °C and 48.6 °C, respectively (Figure S1).

### 2.2. Preparation of AuNPs in the Presence of Dendronized Gelatins with UV Irradiation

**Figure 3:**
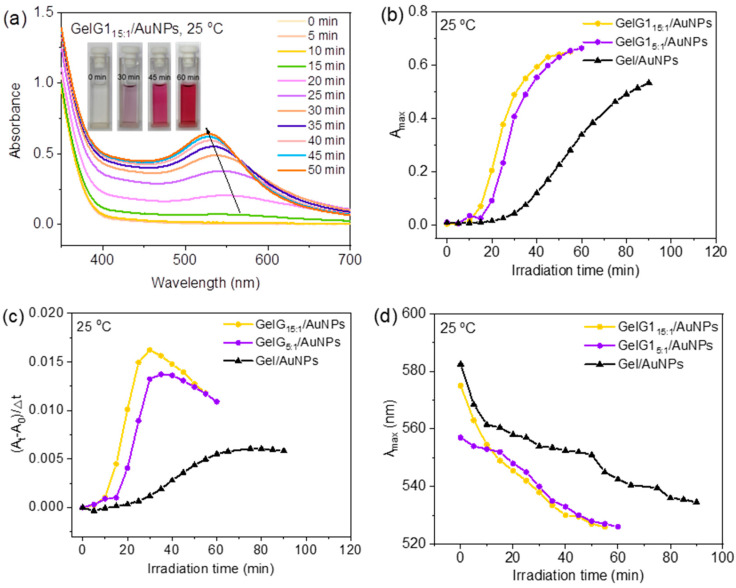
(**a**) UV/vis absorption spectra of AuNPs obtained by in situ reduction of HAuCl_4_ (0.1 mg/mL) with **GelG1_15:1_** (2 mg/mL) through irradiation with UV light (365 nm, 30 W) at 25 °C. Inserts are photographs of aqueous solutions of **GelG1_15:1_** and HAuCl_4_ after UV irradiation for different time. Plots of A_max_ (**b**), reduction rate (**c**) and λ_max_ (**d**) with irradiation time for in situ reduction of HAuCl_4_ (0.1 mg/mL) with **GelG1_15:1_**, **GelG1_5:1_** and gelatin (2 mg/mL) at 25 °C.

Reduction of AuCl_4_^−^ in the presence of **GelG1** at room temperature through ultraviolet irradiation was examined first. Aqueous solution of **GelG1** containing AuCl_4_^−^ changed from colorless to purple after 30 min and to red after 60 min of irradiation (Figure 3a). UV/vis spectra were, therefore, used to follow the reduction process. As shown in Figure 3a, a strong absorption around 520 nm appeared and its intensities increased with irradiation time, corresponding to the surface plasma vibration from AuNP [30]. The plasma resonance peak intensities of the solution increased with irradiation time, indicating that the amount of AuNPs formed by reduction increased with time. For comparison, the reduction of Au(III) in the presence of naked gelatin was performed, and a similar plasma resonance peak appeared with its intensities also increased with irradiation time (Figure S2a). This indicates that the reduction of Au(III) can be conducted in either dendronized gelatins or naked gelatin. We suppose the reduction was driven by phenolic hydroxyl or other reducing groups from the peptide backbone [31]. However, the reduction kinetics are quite different when comparing the naked gelatin to that of **GelG1**. As shown in Figure 3b,c, after irradiation for a certain time, maximum absorbance (A_max_) and reduction rates ((A_t_ − A_0_)/Δt) in the case of **GelG1** are much higher than that for gelatin, suggesting that the dendritic OEGs are supportive to enhance the reduction kinetics. Compared to that from naked gelatin, the wavelength of the maximum absorption (λ_max_) from **GelG1/AuNPs** is blue shifted (Figure 3d), indicating that the formed AuNPs should have smaller sizes. The morphology and sizes of the AuNPs were observed by a transmission electron microscope (TEM). As shown in Figure S2c, uniformed AuNPs with sizes in the range of 8–12 nm were observed from gelatin, while sizes in the range of 5–8 nm were observed from **GelG1** (Figure S2d). The AuNPs formed in the matrix of dendronized gelatins and gelatin were investigated by dynamic light scattering (DLS). The hydrodynamic radii (R_h_s) of **GelG1/AuNPs** and **Gel/AuNPs** at 25 °C are plotted in Figure S2e, and it was found that R_h_ of **GelG1/AuNPs** is approximately 35 nm, which is slightly smaller than that of **Gel/AuNPs** (about 38 nm).

The effect of the grafting ratio of dendritic OEGs on the modified gelatins for the preparation of AuNPs was checked. As shown in Figure 3c and Figure S2b, the reduction rates of both **GelG1_15:1_** and **GelG1_5:1_** were greater than that of the naked gelatin, and **GelG1_15:1_** exhibited the highest rate. The grafting coverage of dendritic OEGs in **GelG1_15:1_** (86.7%) was higher than that of **GelG1_5:1_** (54.4%), suggesting that abundant dendritic OEGs may have provided enhanced microconfinement to shield the solvation of metal anions, resulting in the promotion of the in situ reduction of Au(III) to form AuNPs. Therefore, an increase in the grafting ratio of dendritic OEGs is helpful to create the confined microenvironment to increase the reduction rate.

**Figure 4:**
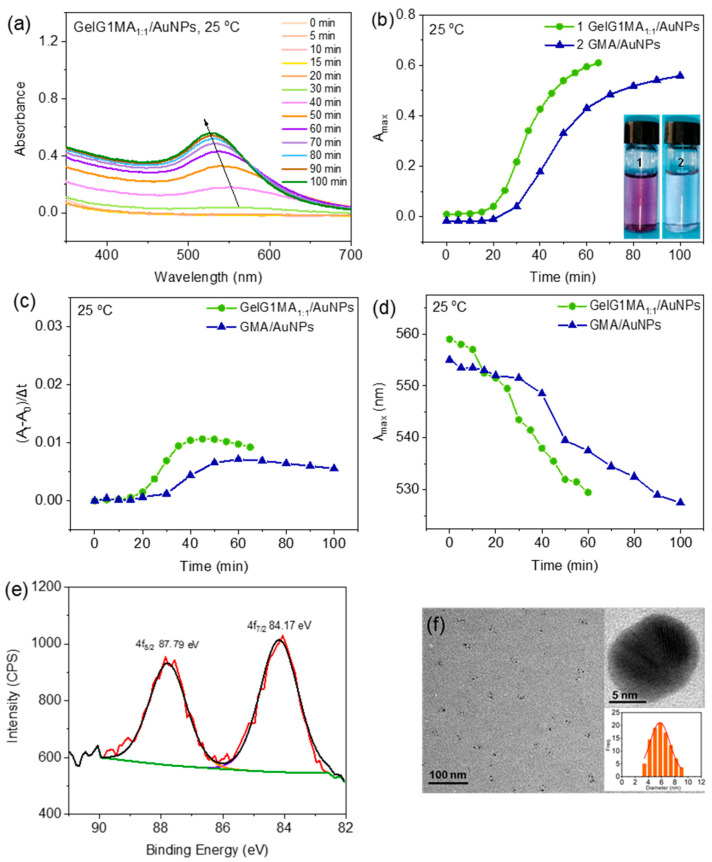
(**a**) UV/vis absorption spectra of AuNPs obtained by in situ reduction of HAuCl_4_ (0.1 mg/mL) with **GelG1MA_1:1_** (2 mg/mL) through irradiation with UV light (365 nm, 30 W) at 25 °C. Plots of A_max_ and photographs (**b**), reduction rate (**c**) and λ_max_ (**d**) with irradiation time for in situ reduction of HAuCl_4_ (0.1 mg/mL) with **GelG1MA_1:1_** and **GMA** (2 mg/mL) at 25 °C. X-ray photoelectron spectroscopy (XPS) spectra (**e**) and TEM photographs and particle size distribution (**f**) of the AuNPs obtained from **GelG1MA_1:1_** (2 mg/mL) under UV irradiation at 25 °C.

Reduction of HAuCl_4_ to form AuNPs was also performed in the presence of **GelG1MA** through UV irradiation. **GelG1MA_1:1_** carried the same dendritic OEGs as **GelG1** but with a slightly lower grafting ratio (45.6%). The reduction process was followed by UV/vis spectroscopy, and the spectra are shown in Figure 4a. Similar to the case from **GelG1**, there was a plasma resonance absorption near 550 nm after UV irradiation and its intensity increased with irradiation time, indicative of forming AuNPs. The aqueous solution of **GelG1MA_1:1_** containing AuCl_4_^−^ changed from colorless to purple after UV irradiation for 60 min at 25 °C (Figure 4b), which also indicates the reduction of Au(III) into Au(0). For comparison, the reduction of AuCl_4_^−^ was also performed in the presence of GMA, where gelatin carries no dendrons but only the methacrylol pendants (Figure S3a), and the solution turned light blue. Compared to the case of **GMA/AuNPs**, the reduction rates for **GelG1MA_1:1_** were higher (Figure 4b) and the maximum absorption from **GelG1MA_1:1_/AuNPs** solution stronger (Figure 4c). These suggest that reduction efficiency for **GelG1MA_1:1_** was significantly enhanced when compared to that for GMA. This again supports that dendritic OEG pendants may have provided a hydrophobic microenvironment through their crowded packing along the gelatin chains, which strengthened the interactions between the polymers and Au(III) to promote the reduction.

Compared to that from GMA, the wavelength of the maximum absorption from **GelG1MA_1:1_/AuNPs** blue shifted (Figure 4d), indicating that the formed AuNPs should be smaller in size. The XPS spectra of the AuNPs were recorded, and the binding energies of the Au (4f_7/2_) and Au (4f_5/2_) peaks appeared at 84.17 eV, 87.79 eV, respectively, which can be assigned to Au(0) (Figure 4e). This further supports the reduction of Au(III) to form Au(0). The morphology and sizes of the AuNPs was further observed by TEM. As shown in Figure 4f, uniformed AuNPs with sizes in the range of 4–8 nm were observed from **GelG1MA_1:1_**. The lattice plane spacing was found to be 2.15 Å, which corresponds to the (111) plane of the face-centered cubic gold crystals [32]. Differently, AuNPs with much larger sizes in the range of 8–12 nm were observed from **GMA** (Figure S3b). As shown in Figure S3c, the R_h_ of **GelG1MA_1__:1_/AuNPs** is approximately 52 nm, slightly smaller than that of **GMA/AuNPs** (about 55 nm). The particle size of AuNPs prepared in the presence of **GelG1MA** is smaller, which may be due to the hydrophobic microenvironment provided by the crowded OEG dendrons. This makes the interaction between the dendronized gelatins and Au(III) stronger, which promotes the reduction. In addition, the steric hindrance effect from the dendrons affords better stability to the formed AuNPs.

### 2.3. Effects of the Thermoresponsive Properties of the Dendronized Gelatins on the Formation of AuNPs

**Figure 5:**
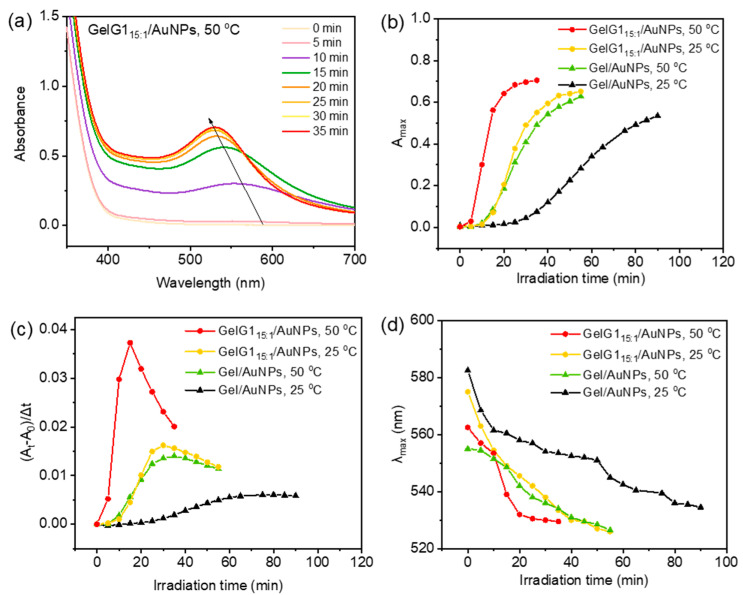
(**a**) UV/vis absorption spectra of AuNPs obtained by in situ reduction of HAuCl_4_ (0.1 mg/mL) with **GelG1_15:1_** (2 mg/mL) at 50 °C, and plots of A_max_ (**b**), reduction rate (**c**) and λ_max_ (**d**) with irradiation time for in situ reduction of HAuCl_4_ (0.1 mg/mL) with **GelG1_15:1_**, **GelG1_5:1_** and gelatin (2 mg/mL) at 50 °C. The data from reduction at 25 °C were also included for comparison.

Since these dendronized gelatins are thermoresponsive, their modulation of the reduction at elevated temperature was examined. The effect of the thermoresponsive properties of dendronized gelatins on the preparation of AuNPs can be investigated by UV irradiation above the cloud point (50 °C), compared to that below the cloud point (25 °C). From the UV/vis spectra in Figure 5a, when irradiated at 50 °C, which is above the T_cp_ of **GelG1**, plasma resonance with much stronger intensities than that at room temperature appeared (Figure 5b). This indicates that reduction kinetics can be greatly enhanced by increases in temperature. To verify whether this enhancement was originated from temperature increase itself or the combined effects from the thermally induced dehydration and collapse of the dendritic OEGs, the reduction of Au(III) at elevated temperature in the presence of naked gelatin, which is not thermoresponsive, was performed and compared (Figure S4). As shown in Figure 5c, reduction kinetics increased in both naked gelatin and **GelG1** with temperature, with the latter more pronounced. This indicates again that crowded dendritic OEGs are supportive to the reduction. Furthermore, the maximum absorption wavelength of AuNPs blue shifted to 525 nm from 575 nm (Figure 5d), indicating that the size of AuNPs gradually decreased at elevated temperature.

**Figure 6:**
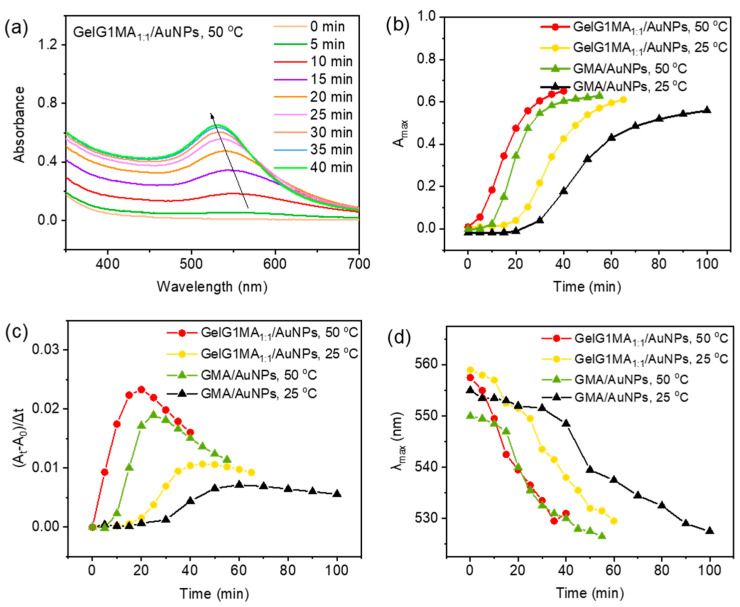
(**a**) UV/vis absorption spectra of AuNPs obtained by in situ reduction of HAuCl_4_ (0.1 mg/mL) with **GelG1MA_1:1_** (2 mg/mL) at 50 °C, and plots of A_max_ (**b**), reduction rate (**c**) and λ_max_ (**d**) with irradiation time for in situ reduction of HAuCl_4_ (0.1 mg/mL) with **GelG1MA_1:1_** and **GMA** (2 mg/mL) at 50 °C. The data from reduction at 25 °C were also included for better comparison.

Similar to the case at room temperature, the aqueous solution of **GelG1MA_1:1_** containing AuCl_4_^−^ irradiation with UV light at 50 °C transformed from a colorless solution into purple, as shown in Figure S5a. From the UV/vis spectra in Figure 6a, when irradiated at 50 °C, which is above the T_cp_ of **GelG1MA_1:1_**, the maximum absorption showed much enhanced intensities at the same UV irradiation time when compared to the case at room temperature (Figure 6b), indicating that the reduction rate was obviously accelerated by the increased temperature. However, the reduction rate of AuNPs by GMA at elevated temperature remained relatively slow, as shown in Figure 6c and Figure S5b. This again suggests that the enhanced hydrophobic microenvironment from the dehydrated dendritic OEGs at elevated temperatures enhanced the reduction. Furthermore, when irradiated at 50 °C, the maximum absorption wavelength of AuNPs blue shifted to 525 nm from 560 nm (Figure 6d), indicating that the size of AuNPs gradually decreased at elevated temperature.

### 2.4. Stability of AuNPs Formed in the Matrices of Dendronized Gelatins

**Figure 7:**
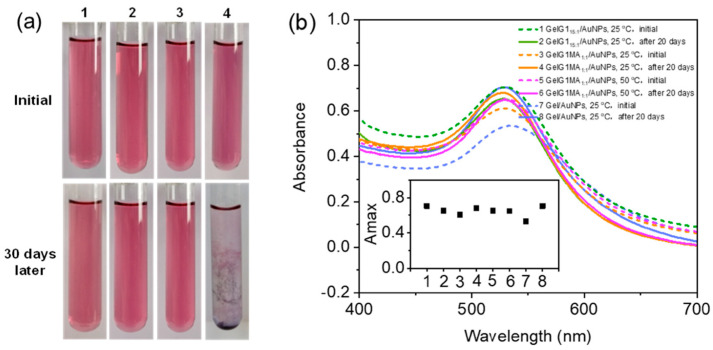
Stability of AuNPs in aqueous solutions. (**a**) Photographs of solutions of **GelG1_15:1_/AuNP**, 25 °C (**1**), **GelG1MA_1:1_/AuNP**, 25 °C (**2**), **GelG1MA_1:1_/AuNP**, 50 °C (**3**), and **Gel/AuNP**, 25 °C (**4**). (**b**) UV/vis spectra of the polymer/AuNPs solutions stored over different periods at room temperature. The inset shows the maximum absorbance with storage time.

The above results show that dendronized gelatins can reduce Au(III) to form well-defined AuNPs through UV irradiation. This method does not need the addition of any external reducing agent. To explore the feasibility of the methodology developed in present work, the stability of the AuNPs formed in the matrix of dendronized gelatins were investigated. After storage at 25 °C for 30 days, the pink solutions from **GelG1_15:1_/AuNPs** or **GelG1MA_1:1_/AuNPs** remained clear and homogeneous, as shown in Figure 7a, indicating their high stability. In contrast, **Gel/AuNPs** became turbid and precipitation was observed. UV/vis spectroscopy was also applied to follow the stability of the AuNPs. After 20 days of storage at room temperature, A_max_ from the dendronized gelatins did not change significantly (Figure 7b), while A_max_ from the **Gel/AuNPs** showed the most obvious change. The above results indicate that dendronized gelatins act as reducing agents for the transformation of Au(III) into Au(0), and at the same time, as a stabilizer to the formed nanoparticles. The electrostatic or coordination interactions between the dendronized gelatins and the formed AuNPs should have contributed to their stability in aqueous media. On the other hand, crowding from the dendritic units in the dendronized gelatins may have also provided an enveloping effect to prevent the AuNPs from agglomeration, making them dispersed with high stability [28].

### 2.5. Fabrication of Dendronized Gelatin/AuNP Hydrogels

**Figure 8:**
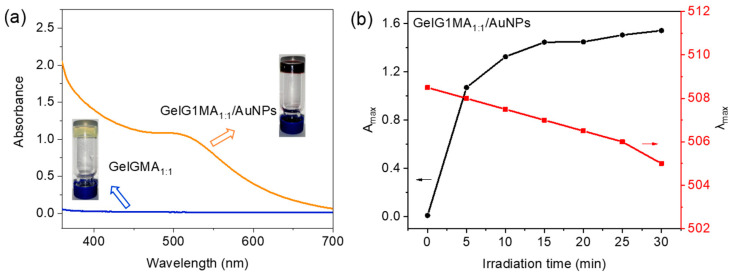
(**a**) UV/vis absorption spectra and photographs of **GelG1MA_1:1_/AuNPs** and **GelG1MA_1:1_** hydrogel after UV irradiation for 5 min. (**b**) Plots of A_max_ and λ_max_ of **GelG1MA_1:1_/AuNPs** hydrogel with UV irradiation time.

**GelG1MA** contains unsaturated double bonds. Through UV irradiation, they can act as a matrix for in situ reduction to form AuNPs, and simultaneously transform into hydrogels via photo-crosslinking polymerization (Scheme S2). Therefore, a mixture of **GelG1MA_1:1_** (10 wt%), HAuCl_4_ (2.5 mmol/L) and photo-initiator 2959 (2 wt%) was illuminated by ultraviolet light (30 W, 365 nm), which changed from colorless solution into dark purple hydrogel (Figure 8a). For comparison, **GelG1MA_1:1_** hydrogel was prepared by the same method without HAuCl_4_, which exhibited a light-yellow color. Both hydrogels can keep their shape when heated to 50 °C, indicating chemical crosslinking. The reduction of HAuCl_4_ in the presence of **GelG1MA_1:1_** to form **AuNP**s and transform into hydrogel was traced by UV/vis spectroscopy. The plasma resonance absorption peak of AuNPs appeared at 520 nm after UV irradiation for 5 min (Figure 8a), and the intensity of the absorption peak remained unchanged after about 15 min (Figure 8b), indicating that **GelG1MA_1:1_** had reduced most of Au(III) into AuNPs at that condition. The λ_max_ exhibited a blue shift to 505 nm from about 508, indicating that the elongation of irradiation afforded AuNPs with slightly smaller sizes. The dendronized gelatin/gold nanoparticle composite hydrogels were successfully obtained through in situ reduction and photo crosslinking polymerization, prompted by UV irradiation. This method has the advantages of fast photo crosslinking to form hydrogels and the high efficiency of Au(III) reduction into gold nanoparticles. This work provides a simple, rapid and in situ reduction method for the preparation of gold nanoparticles’ composite hydrogels, which may have broad application prospects in biomedical fields such as bioimaging, biosensing and drug delivery. We believe these thermoresponsive dendronized gelatin hydrogel combined AuNP composites could be important in promising bioapplications.

## 3. Materials and Methods

### 3.1. Materials

Dendronized gelatins **GelG1**s were prepared as described previously [29]. Gelatin was purchased from Sigma-Aldrich Chemical Co., Shanghai, China. Tetrachloroauric hydrate hydrochloride, 2-hydroxy-4’-(2-hydroxyethoxy)-2-methylpropiophenone, and ethylene diamine tetraacetic acid were purchased from Sinopharm Chemical Reagent Co., Ltd. (Shanghai, China). Phosphate-buffered saline (PBS, pH 7.4) containing 0.0067 M phosphate without calcium and magnesium was purchased from Hyclone Thermo Fisher (Logan, QLD, USA). Other reagents and solvents were purchased at reagent grade and used without further purification.

### 3.2. Instrumentation and Measurements

^1^H NMR spectra were recorded on a Bruker AV 500 (500 MHz) spectrometer. UV/vis measurements were conducted on a JASCO V-750 spectrophotometer equipped with a thermostatically regulated bath. Aqueous polymer solutions were placed in the spectrophotometer (path length 1 cm). Fluorescence (FL) spectra were recorded in a fluorescence spectrometer (Horiba Jobin Yvon Fluorolog-3) equipped with a temperature control device. Dynamic light scattering (DLS) measurements were performed on a DynaPro Nanostar. X-ray photoelectron spectroscopy (XPS) spectra were recorded on an AXIS Supra+ (Shimadzu, Manchester, UK). This system uses a focused monochromatic Al K_α_ X-ray (1486.7 eV) source for excitation. The binding energies were referenced to C1s at 284.8 eV from hydrocarbon to compensate for the charging effect. The micromorphology of in situ-formed spherical AuNPs was imaged by using a field-transmission electron microscope (JEM-2100, INCAX-Max80) with an accelerating voltage of 160 kV. Sample preparation: An appropriate amount of solution was taken into the centrifuge tube, and centrifuged with a rotating speed 10,000 RPM for 15 min. The supernatant was discarded, and ultrapure water was added to the ultrasonic dispersion and centrifuged twice. After discarding the supernatant, absolute ethanol was added to the ultrasonic dispersion, which was then dispersed on copper wire.

### 3.3. Synthesis of GelG1MA

**GelG1** (5 g) was dissolved in Dulbecco’s phosphate-buffered saline (DPBS) solution (50 mL) at 50 °C, followed by the addition of methacrylic anhydride (MA) at a rate of 0.5 mL/min. The molar ratio of the amino group from gelatin to MA was set to be 1:20. After stirring for 4 h, the reaction mixture was diluted with DPBS and dialyzed for 7 days against distilled water with dialysis membranes (MWCO 12–14 kD). Freeze-drying afforded the product as a white solid with a yield of 48%. The structure of **GelG1MA_1:1_** was confirmed by NMR spectroscopy. ^1^H NMR (500 MHz, D_2_O): δ = 1.28–1.45 (br, CH_3_), 3.67–3.95 (br, CH_2_), 4.31–4.44 (br, CH_2_), 5.19 (s, CH_2_), 5.79 (br, C=CH_2_), 6.18 (br, C=CH_2_) 6.94 (s, Ar–H).

### 3.4. Fabrication of AuNPs in the Matrix of Gelatin or Modified Gelatins

Gelatin or modified gelatins (4 mg) were mixed with HAuCl_4_·4H_2_O (0.2 mg) in aqueous solution (1 mL). The mixture was stirred by a vortex oscillator, then irradiated by ultraviolet light (365 nm, 30 W) for a designed time period (60–90 min). The distance between the lamp and the sample was fixed at 10 cm. UV/vis spectroscopy was applied to follow the reduction process. AuNPs obtained from the matrix of naked gelatin, gelatin methacryloyl, dendronized gelatin, and dendronized gelatin methacryloyl are named as **Gel/AuNPs**, **GMA/AuNPs**, **GelG1/AuNPs**, and **GelG1MA/AuNPs**, respectively.

### 3.5. Fabrication of GelG1MA/AuNP Hydrogel

The mixture of **GelG1MA_1:1_** (100 mg/mL), HAuCl_4_ (1 mg/mL) and photo-initiator 2959 (20 mg/mL) was oscillated for 30 s in the scroll oscillator to form a uniformly mixed solution. The mixture was illuminated for 30 min by ultraviolet lamp (30 W, 365 nm), and the mixture changed from a colorless solution into a dark purple hydrogel.

## 4. Conclusions

We have developed a green and convenient method in the present work to generate stable AuNPs by using dendronized gelatins or dendronized gelatin methacrylates as precursors, triggered by UV irradiation. The reduction can be processed efficiently without any additional reducing chemicals. Dendronized gelatins act as both a reducing agent for the transformation of Au(III) into Au(0) and a stabilizer for the formed nanoparticles through crowded dendritic OEG units. The dendritic OEGs provide a hydrophobic microenvironment to promote the formation of AuNPs, and the reduction rate can be enhanced with an increase in grafting coverage of the dendritic OEGs, as well as the thermally induced aggregation of polymer chains. The high stability of the nanoparticles in the matrix of dendronized gelatins has been verified through long-term storage. Therefore, the formation of AuNPs within the envelope of dendronized gelatins avoids cumbersome operation and environmental pollution. Dendronized gelatin/AuNP composite hydrogels can be readily prepared from dendronized gelatin methacryloyl via UV irradiation through simultaneous reduction and photo-crosslinking polymerization. This clean and environmentally friendly method for the synthesis of AuNPs may be extended for the fabrication of other noble metal nanoparticles and shed light on promising applications in nano biosensors and nano devices.

## Data Availability

Not applicable.

## Supplementary Materials

The following supporting information can be downloaded at: https://www.mdpi.com/article/10.3390/molecules27186096/s1. Scheme S1: Synthetic procedures for dendronized gelatin **GelG1MA**. Reagents and conditions: methacryloyl anhydride, **GelG1**, water, 50 °C, 4 h (88%); Scheme S2: Preparation of GelG1MA hydrogel by UV irradiation; Figure S1: Plots of transmittance vs. temperature for **GelG1_15:1_**, **GelG_15:1_** and **GelG1MA_1:1_** in water. Polymer concentration = 0.5 wt%. Heating rate = 0.2 °C/min; Figure S2: UV/vis absorption spectra of gold nanoparticles obtained by in situ reduction of HAuCl_4_ under UV irradiation at 25 °C in the presence of gelatin (**a**) and **GelG1_5:1_** (**b**). TEM photographs of the AuNPs obtained through UV irradiation at 25 °C from gelatin (**c**) and **GelG1_5:1_** (**d**). Hydrodynamic radii of nanoparticles (intensity) obtained by in situ reduction of HAuCl_4_ by **GelG1_5:1_** and gelatin through UV irradiation at 25 °C (**e**). Insets in (**c**,**d**) are particle size distribution of the nanoparticles. HAuCl_4_ concentration = 0.1 mg/mL. Polymer concentration = 2 mg/mL; Figure S3: UV/vis absorption spectra of gold nanoparticles obtained by in situ reduction of HAuCl_4_ with **GMA** through UV irradiation at 25 °C (**a**). TEM photographs of AuNPs obtained from **GMA** through UV irradiation at 25 °C (**b**). Hydrodynamic radii of nanoparticles (intensity) obtained by in situ reduction of HAuCl_4_ by **GelG1MA_1:1_** and **GMA** through UV irradiation at 25 °C (**c**). Inset in (**b**) is particle size distribution of the nanoparticles. HAuCl_4_ concentration = 0.1 mg/mL. Polymer concentration = 2 mg/mL; Figure S4: UV/vis absorption spectra of gold nanoparticles obtained by in situ reduction of HAuCl_4_ (0.1 mg/mL) with naked gelatin (2 mg/mL) under different UV irradiation time at 50 °C; Figure S5: (**a**) Photographs of aqueous solutions of **GelG1MA_1:1_** (2 mg/mL) and GMA (2 mg/mL) with HAuCl_4_ (0.1 mg/mL) after irradiated by UV light (365 nm, 30 W) after 30 min at 50 °C (Bottle 1: **GelG1MA_1:1_/AuNPs**, bottle 2: **GMA/AuNPs**). (**b**) UV/vis absorption spectra of gold nanoparticles obtained by in situ reduction of HAuCl_4_ (0.1 mg/mL) with **GMA** (2 mg/mL) under different UV irradiation time at 50 °C.

## Sample Availability

Samples of the compounds **GelG1** and **GelG1MA** are available from the authors.
