# Supplementary material for: Dendronized Gelatin-Mediated Synthesis of Gold Nanoparticles
Source: Molecules. 2022 Sep 18;27(18):6096. doi: 10.3390/molecules27186096 (PMC9500934; doi:10.3390/molecules27186096)
Supplement: Supplementary file 1 [file molecules-27-06096-s001.zip › molecules-1900378-supplementary.pdf]

Supporting Information

Dendronized Gelatins Mediated Synthesis of Gold Nanoparticles

Yan Ding[1,2], Xiacong Zhang[1], Wen Li[1,*], and Afang Zhang[1]

[1] International Joint Laboratory of Biomimetic and Smart Polymers, School of Materials Science & Engineering, Shanghai University, Shanghai 200444, China

[2] Institute of Chemistry and Materials Science, Huaibei Normal University, Dongshan Road 100, Huaibei 235000, China

Table of Contents

Scheme S1. Synthetic procedures for dendronized gelatins **GelG1MA**. Reagents and conditions: methacryloyl anhydride, 50 °C, 4 h (88%) 4

Figure S1. Plots of transmittance vs temperature for **GelG1$_{15:1}$**, **GelG$_{15:1}$** and **GelG1MA$_{1:1}$** in water. (C = 0.5 wt%; Heating rate = 0.2 ºC/min...4

Figure S2. UV/vis absorption spectra of gold nanoparticles obtained by *in situ* reduction of $HAuCl_4$ under UV irradiation at 25 ºC in the presence of gelatin (a) and **GelG1$_{5:1}$** (b). TEM photographs of the AuNPs obtained through UV irradiation at 25 ºC from gelatin (c) and **GelG1$_{5:1}$** (d). Hydrodynamic radii of nanoparticles (intensity) obtained by *in situ* reduction of $HAuCl_4$ by **GelG1$_{5:1}$** and gelatin through UV irradiation at 25 ºC (e). Insets in c) and d) are particle size distribution of the nanoparticles...5

Figure S3. UV/vis absorption spectra of gold nanoparticles obtained by *in situ* reduction of $HAuCl_4$ with **GMA** through UV irradiation at 25 ºC (a). TEM photographs of AuNPs obtained from **GMA** through UV irradiation at 25 ºC (b). Hydrodynamic radii of nanoparticles (intensity) obtained by *in situ* reduction of $HAuCl_4$ by **GelG1MA$_{1:1}$** and **GMA** through UV irradiation at 25 ºC (c). Inset in b) is particle size distribution of the nanoparticles..6

Figure S4. UV/vis absorption spectra of gold nanoparticles obtained by in situ reduction of $HAuCl_4$ (0.1 mg/mL) with naked gelatin (2 mg/mL) under different UV irradiation time at 50 °C 7

Figure S5. (a) Photographs of aqueous solutions of **GelG1MA$_{1:1}$** (2 mg/mL) and GMA (2 mg/mL) with $HAuCl_4$ (0.1 mg/mL) after irradiated by UV light (365 nm, 30W) after 30 min at 50 ºC (Bottle 1: **GelG1MA$_{1:1}$/AuNPs**, bottle 2: **GMA/AuNPs**). (b) UV/vis absorption spectra of gold nanoparticles obtained by in situ reduction of $HAuCl_4$ (0.1 mg/mL) with **GMA** (2 mg/mL) under

different UV irradiation time at 50 °C. 7

Scheme S2. Preparation of **GelG1MA** hydrogel by UV irradiation...8

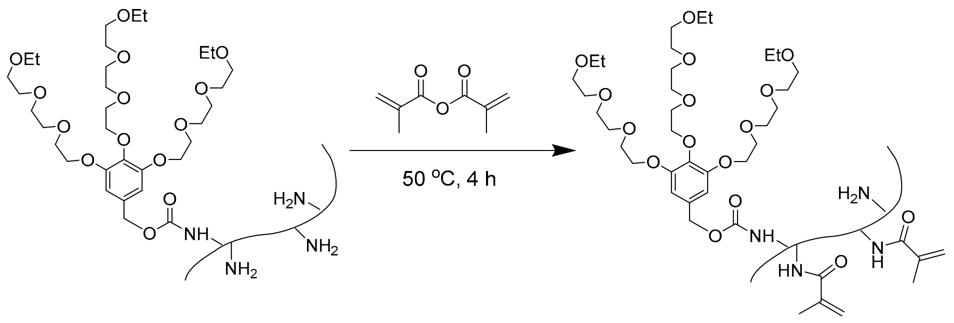

GelG1 **GelG1MA**

Scheme S1. Synthetic procedures for dendronized gelatin **GelG1MA**. Reagents and conditions: methacryloyl anhydride, **GelG1**, water, 50 °C, 4 h (88%).

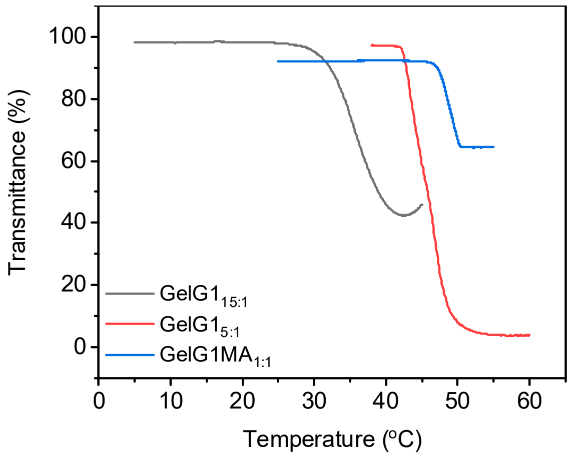

Figure S1. Plots of transmittance vs temperature for **GelG1$_{15:1}$**, **GelG$_{15:1}$** and **GelG1MA$_{1:1}$** in water. Polymer concentration = 0.5 wt%. Heating rate = 0.2 ºC/min.

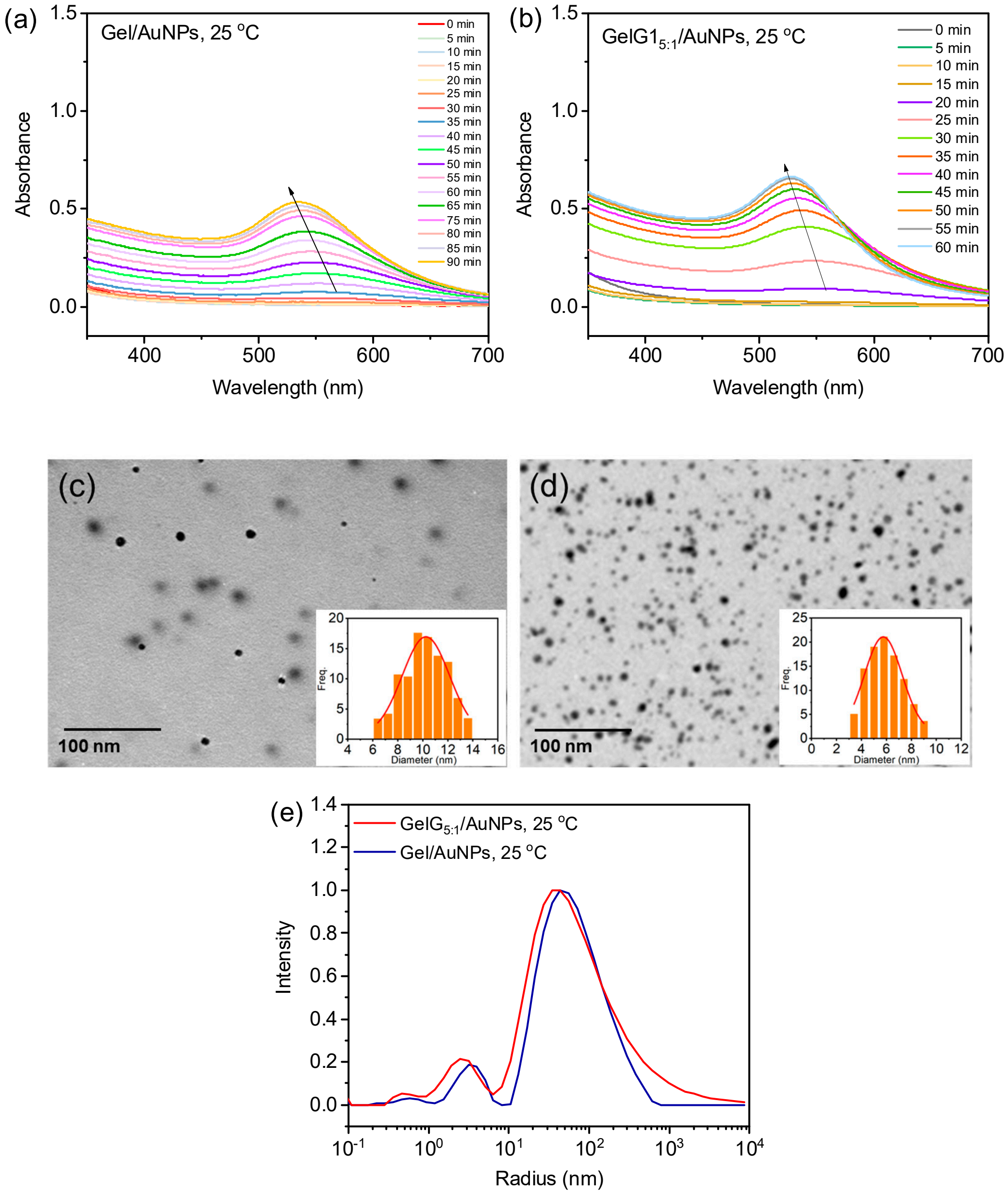

Figure S2. UV/vis absorption spectra of gold nanoparticles obtained by *in situ* reduction of $HAuCl_4$ under UV irradiation at 25 ^{o}C in the presence of gelatin (a) and **GelG1$_{5:1}$** (b). TEM photographs of the AuNPs obtained through UV irradiation at 25 ^{o}C from gelatin (c) and **GelG1$_{5:1}$** (d). Hydrodynamic radii of nanoparticles (intensity) obtained by *in situ* reduction of $HAuCl_4$ by **GelG1$_{5:1}$** and gelatin through UV irradiation at 25 ^{o}C (e). Insets in c) and d) are particle size distribution of the nanoparticles. $HAuCl_4$ concentration = 0.1 mg/mL. Polymer concentration = 2 mg/mL.

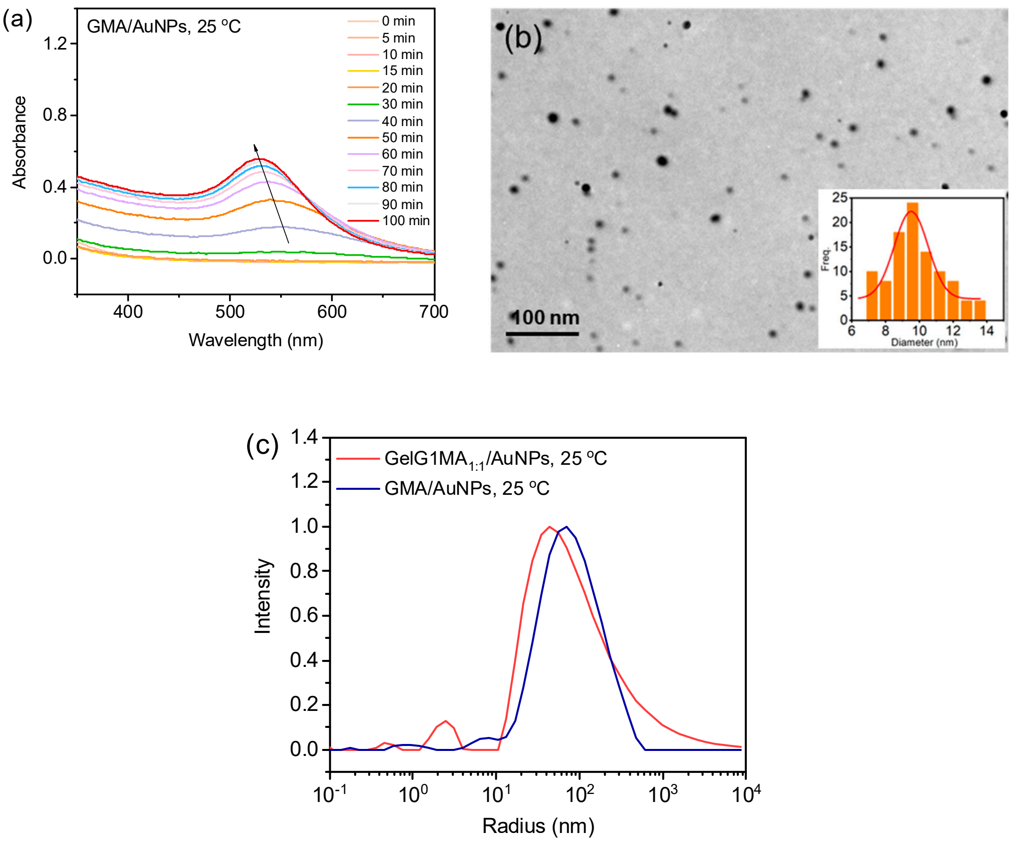

Figure S3. UV/vis absorption spectra of gold nanoparticles obtained by *in situ* reduction of $HAuCl_4$ with **GMA** through UV irradiation at 25 °C (a). TEM photographs of AuNPs obtained from **GMA** through UV irradiation at 25 °C (b). Hydrodynamic radii of nanoparticles (intensity) obtained by *in situ* reduction of $HAuCl_4$ by **GelG1MA$_{1:1}$** and **GMA** through UV irradiation at 25 °C (c). Inset in b) is particle size distribution of the nanoparticles. $HAuCl_4$ concentration = 0.1 mg/mL. Polymer concentration = 2 mg/mL.

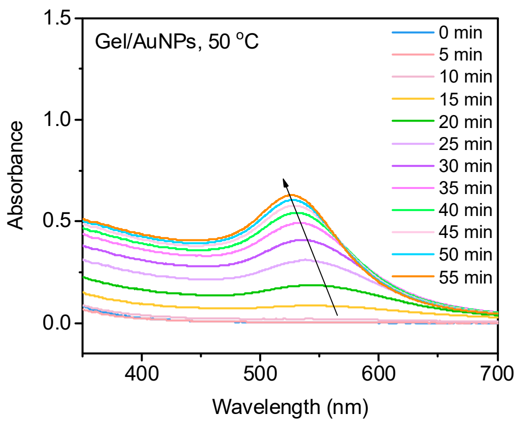

Figure S4. UV/vis absorption spectra of gold nanoparticles obtained by in situ reduction of $HAuCl_4$ (0.1 mg/mL) with naked gelatin (2 mg/mL) under different UV irradiation time at 50 °C.

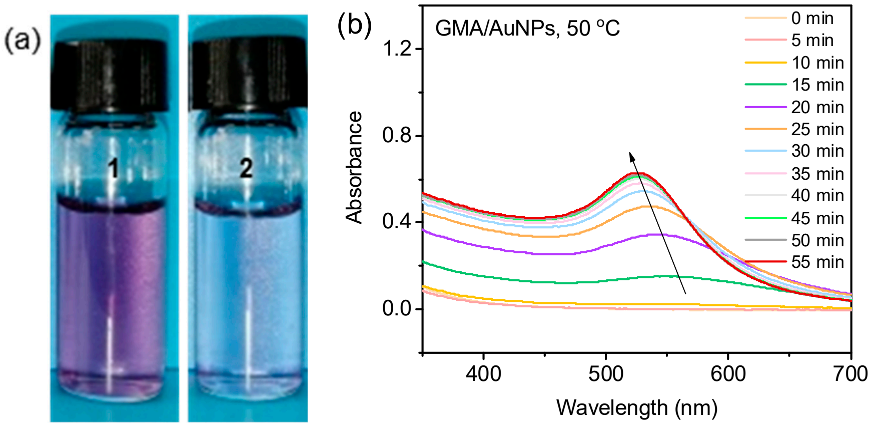

Figure S5. (a) Photographs of aqueous solutions of **GelG1MA$_{1:1}$** (2 mg/mL) and GMA (2 mg/mL) with $HAuCl_4$ (0.1 mg/mL) after irradiated by UV light (365 nm, 30W) after 30 min at 50 °C (Bottle 1: **GelG1MA$_{1:1}$/AuNPs**, bottle 2: **GMA/AuNPs**). (b) UV/vis absorption spectra of gold nanoparticles obtained by in situ reduction of $HAuCl_4$ (0.1 mg/mL) with **GMA** (2 mg/mL) under different UV irradiation time at 50 °C.

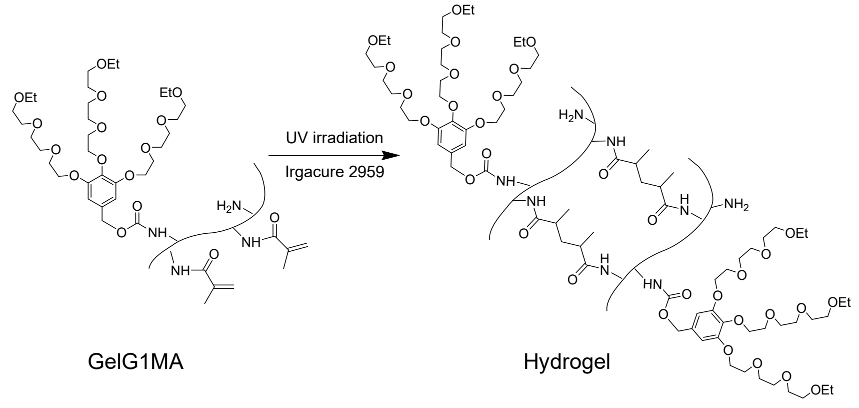

Scheme S2. Preparation of GelG1MA hydrogel by UV irradiation.
